# Structure, regulation, and mechanisms of nonmuscle myosin-2

**DOI:** 10.1007/s00018-024-05264-6

**Published:** 2024-06-15

**Authors:** Krishna Chinthalapudi, Sarah M. Heissler

**Affiliations:** Department of Physiology and Cell Biology, Dorothy M. Davis Heart and Lung Research Institute, The Ohio State University College of Medicine, Columbus, OH 43210 USA

**Keywords:** Myosin, Cytoskeleton, Enzymology, Structure, Mechanoenzyme, Molecular motor

## Abstract

Members of the myosin superfamily of molecular motors are large mechanochemical ATPases that are implicated in an ever-expanding array of cellular functions. This review focuses on mammalian nonmuscle myosin-2 (NM2) paralogs, ubiquitous members of the myosin-2 family of filament-forming motors. Through the conversion of chemical energy into mechanical work, NM2 paralogs remodel and shape cells and tissues. This process is tightly controlled in time and space by numerous synergetic regulation mechanisms to meet cellular demands. We review how recent advances in structural biology together with elegant biophysical and cell biological approaches have contributed to our understanding of the shared and unique mechanisms of NM2 paralogs as they relate to their kinetics, regulation, assembly, and cellular function.

## Introduction

Members of the myosin superfamily are ubiquitous actin-based molecular motors in eukaryotes. In humans, the protein products of the 39 myosin genes are grouped into 12 distinct classes. The largest class of myosins is referred to as class-2 and consists of 10 sarcomeric myosin-2 paralogs, one smooth muscle myosin-2 paralog, and three nonmuscle myosin-2 (NM2) paralogs at the protein level [1, 2]. The three NM2s (NM2A, NM2B, NM2C) are expressed from three genes (*MYH9, MYH10, MYH14*) in a tissue-specific and developmentally-dependent manner [3, 4]. NM2 paralogs are phylogenetically more closely related to smooth muscle myosin-2 than to sarcomeric myosin-2s [5]. NM2 genes are broadly expressed and their gene products have been implicated in countless fundamental processes with particular relevance to cell adhesion, division, and migration [3, 6]. NM2 paralogs have diverse and specialized roles in cells, share highly conserved structural features, and generate mechanical force by pulling on antiparallel actin filaments. Thereby, they directly influence actin organization, tension, elasticity, mechanoresponsiveness, and other cellular properties [7–9]. NM2 paralogs have balanced spatially and temporally determined functions, each contributing slightly differently to specialized cellular processes [3, 10–14]. Compared to NM2A and NM2B which are abundantly present (~ 0.2–10.8 µM NM2A, ~ 0.036–0.68 µM NM2B) in most cells and tissues, NM2C is present in a limited number of cells and tissues and typically in less quantities (~ 0.049 µM) [15–17]. Cells are exquisitely sensitive to altered NM2 function, regulation, assembly, and expression levels and each of these factors can contribute to disease pathogenesis [4, 18–20]. Because of their widespread importance, NM2s have been the focus of intensive foundational and clinical research efforts in recent years but the molecular mechanisms that link NM2 structure and regulation to its physiological and pathophysiological functions remain largely unexplored [21–23]. This review primarily focuses on mammalian NM2s and their structure, regulation, and mechanisms. While mammalian and lower eukaryotic NM2s exhibit a high degree of structural similarity, their regulation mechanisms frequently diverge. Please note that our scope does not encompass a comprehensive discussion of myosin regulation mechanisms in lower eukaryotes.

## Cellular function and disease

The understanding of the emergent and multifaceted roles of NM2 paralogs in essential cellular processes including cell division, migration, differentiation, and adhesion was greatly facilitated through the generation of various animal models in conjunction with elegant cell biological studies. Mouse models revealed specific as well as redundant functions for NM2 paralogs during development. The comparison of NM2 expression patterns in the developing mouse embryo shows a broad tissue distribution at E11.5 with paralog-specific enrichments in organs, tissues, and cell types [4]. For example, NM2A is enriched in the vasculature, NM2B in neurons, and NM2C in the pituitary gland in the brain [4]. NM2A and NM2C are enriched in epithelial cells whereas NM2B is enriched in serosal cells of the intestine at E16.5 [4, 5]. Ablation of NM2A or NM2B in mice is lethal at E6.5 and E14.5, respectively [4, 14, 24, 25]. Mice ablated for NM2C show no obvious defects and survive to adulthood [16]. Ablation of NM2A in mice results in defects in the development of the placenta that cannot be rescued by the expression of NM2B from the *Myh9* locus [4, 11]. Animal models also showed that NM2B is essential for the development of the heart. Ablation of NM2B reduces the proliferation and causes the misalignment of cardiac myocytes [4, 16, 24, 26]. At the cellular level, NM2B, but not NM2C, has been shown to play key roles in cell division in normal cells. Small interfering RNA knockdown of NM2B results in multinucleated cells in vitro and knockout of NM2B in a mouse model results in a ~ 70% decrease in the number and in an increase in binucleation of cardiac myocytes from 1 to 23% compared to control myocytes by E12.5 [16, 26, 27]. The detailed mechanisms of NM2 paralogs play in diverse cellular processes have been reviewed in detail elsewhere [3, 28–30].

Mutations in genes encoding NM2 paralogs have been associated with human diseases. Heterozygous mutations in *MYH9* cause *MYH9*-related disorders (*MYH9*-RD) that are characterized by macrothrombocytopenia with or without granulocyte inclusions, nephritis, or sensorineural hearing loss [31]. Further, mutations in *MYH9* have been associated with autosomal dominant deafness (DFNA17) [32]. Interestingly, mice harboring *MYH9*-RD point mutations exhibit anomalies associated with the disease but also cause male infertility, which is unreported in humans [19, 33]. Mutations in *MYH14* have been associated with peripheral neuropathy, myopathy, hoarseness, and hearing loss (PNMHH) in addition to autosomal dominant nonsyndromic deafness (DFNA4A) [31]. More recently, a link between heterozygous mutations in *MYH10* and neurodevelopmental disorders as well as congenital abnormalities in most organ systems has been proposed [34, 35].

## Domain architecture

**Fig. 1:**
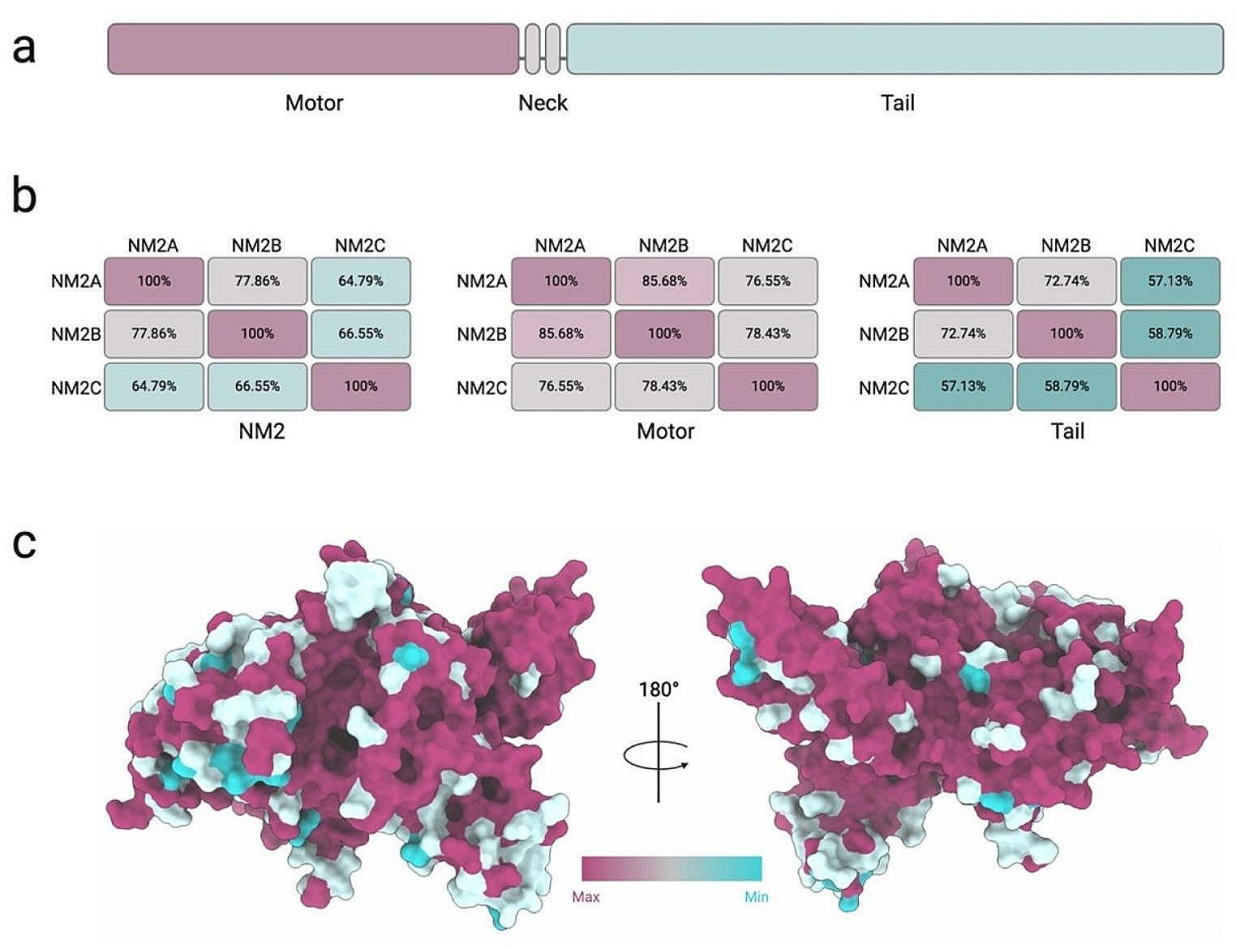
Heavy chain domain organization and structural conservation of human NM2 paralogs **a**. Heavy chain domain organization of a generic human NM2 paralog featuring an N-terminal motor domain (pink), a central neck domain that contains two IQ motifs (grey), and a C-terminal tail domain (green). Schematic not drawn to scale. **b**, Percent identity matrix summarizes the degree of identity between the heavy chains of human NM2 paralogs (left), the motor domain (middle) and the tail domain (right). **c**, Consurf analysis [197] shows the conservation of amino acid positions in the structure of the NM2C motor domain (PDB ID: 5I4E). Views are rotated by 180º

**Fig. 2:**
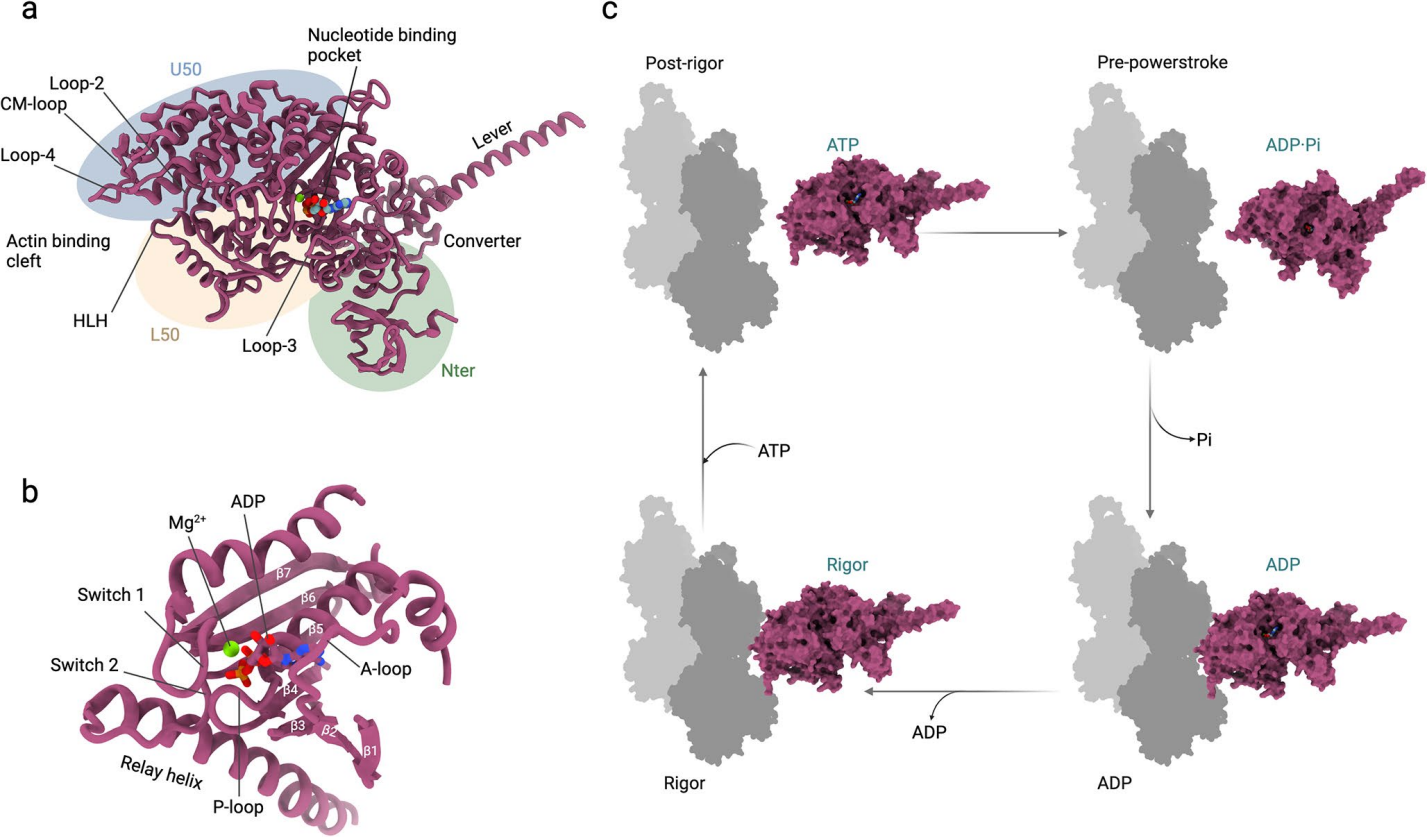
Structural features of the motor domain and kinetic cycle of the NM2s **a**. Key structural features of the myosin motor domain of NM2 paralogs (PDB ID: 5I4E). The myosin motor domain is shown in cartoon representation, the bound nucleotide in spheres. Mg^2+^ is shown as green sphere. Light chains are not shown for clarity. **b**, Key structural features in the nucleotide binding pocket of NM2 paralogs (PDB ID: 5I4E). The myosin motor domain is shown in cartoon representation, the bound nucleotide in stick representation, and Mg^2+^ is shown as green sphere. **c**, Allocation of structural states to the minimal kinetic scheme of the actomyosin ATPase cycle. The myosin motor domain (pink) is shown in surface representation, actin is shown in flat surface representation (grey). Light chains are not shown for clarity. Schematic not drawn to scale

NM2s have a modular domain organization and share common structural characteristics at the protein level [36, 37]. The myosin heavy chain contains an amino-terminal motor domain, a central neck domain, and a carboxy-terminal tail domain (Fig. 1a). NM2 paralogs share a high overall sequence identity (~ 65–78%) with ~ 77–86% sequence identity in the motor domain and ~ 57–73% in the tail (Fig. 1b, c). The motor is a globular ~ 15 nm domain that represents the minimal enzymatically active unit (Fig. 2a). The motor domain contains a nucleotide binding pocket and a binding region for filamentous actin (Fig. 2a, b) [38]. The neck domain contains two IQ motifs that are binding sites for the essential light chain (ELC) and the regulatory light chain (RLC) [39]. The neck domain together with the bound light chains functions as a lever arm during the myosin mechanoenzymatic cycle. Myosin light chains are small EF-hand proteins that confer stabilizing and regulatory functions and possibly directly modulate the enzymatic activity of myosin-2 paralogs [40, 41]. To this date, it remains largely unclear which ELC and RLC isoforms are bound to the NM2 heavy chain in cells [40].

The myosin tail provides the structural framework for the self-assembly of two myosin heavy chains and the assembly of NM2 paralogs into filaments [36]. The tail folds into an extended ~ 160 nm long α-helical structure and ends in a short non-helical tailpiece (NHT). The α-helical tail forms a coiled-coil through hydrophobic interactions that direct the self-assembly of two myosin heavy chains and the bound light chains into an elongated hexameric complex of ~ 525 kDa that is referred to as the monomer. The monomer is the basic building block of NM2 and can further assemble into filaments as well as higher-order filament stacks, as discussed below [42–47].

Mutations have been reported for all human NM2 heavy chain genes and linked to a range of debilitating diseases including blood and kidney disorders, hearing loss, infertility, neurodevelopmental disorders, and cancer [4, 19, 48, 49]. Although the functional consequences of most mutations have not been explored at the protein level, the trend is that mutations in the motor domain decrease enzyme activity while mutations in the tail may alter the assembly properties of NM2 paralogs [50, 51]. Notably, mutations have not been reported in the genes encoding for the NM2 ELC and RLC, unlike in sarcomeric light chain genes where mutations have been linked to different types of cardiac and skeletal myopathies [52].

### Mechanoenzymatic cycle

NM2s convert chemical energy into mechanical force by coupling structural changes in the myosin motor domain to a conserved enzyme cycle (Fig. 2c) [53, 54]. In this mechanoenzymatic cycle, ATP is the substrate, and actin serves as a nucleotide exchange factor for myosin. Per one molecule of ATP hydrolyzed, actin is displaced one discrete distance past myosin. Four major structural states can be distinguished as myosin transitions through its mechanoenzymatic cycle as revealed by crystallographic, negative staining electron microscopy, cryo-electron microscopy (cryo-EM), and kinetic studies (Fig. 2c). In the nucleotide-free rigor state, NM2 tightly binds to actin. The rigor state has higher affinity for actin than the myosin ·ATP state [55–57]. Therefore, ATP binding to the actin-bound myosin motor domain causes the dissociation of myosin from actin. Myosin hydrolyzes ATP to ADP and P_i_ while it undergoes a conformational change, the recovery stroke, that puts the motor in a primed conformation [58]. The ADP·P_i_ state is a stable intermediate with weak actin affinity. Myosin rebinds actin and a conformational change in myosin results in the relative ~ 7 nm displacement of both proteins [59]. The powerstroke is associated with the release of P_i_ but it is still controversial whether both events occur simultaneously or not [60, 61]. ADP is released at the end of the powerstroke and concludes the cycle.

Kinetic studies on single-headed myosin fragments showed that NM2 paralogs and their isoforms have unique kinetic profiles associated with their individual mechanoenzymatic cycles. NM2s are classified as strain sensors and are amongst the slowest myosins that have been kinetically characterized in vitro [55–57, 62–66]. A key feature of the mechanoenzymatic cycle of NM2 paralogs is a low basal ATPase activity that is marginally activated by actin under steady-state conditions, resulting in a poor catalytic efficiency (k_cat_/K_app_) compared to other myosin-2s [55–57, 67]. A kinetic signature of NM2 paralogs is that actin binding does not significantly accelerate the release of ADP from the nucleotide binding pocket [55–57]. This contributes to small kinetic (k_-AD_/k_-D_) and weak thermodynamic (K_AD_/K_D_) coupling ratios. Therefore, ADP binding does not significantly change the actin affinity of NM2s and vice versa [68]. The low actin-activated ADP release is thought to contribute to a slow in vitro sliding velocity [55, 59, 65]. Together, these kinetic signatures result in a duty ratio that is higher compared to that of other myosin-2 paralogs, meaning that NM2s spend a larger amount of time during their enzyme cycle strongly bound to actin [63].

NM2s have a well-defined kinetic behavior in the monomeric form but a very complex collective behavior once they are assembled into filaments. Strikingly, the number of monomers in an NM2 filament is reciprocal to the duty ratio. This ensures that a collective duty ratio > 0.8, which is required for robust motion, is achieved and allows NM2 filaments to move processively and to generate force and movement in cells [59, 65, 69, 70]. The lower duty ratio of NM2A compared to NM2B likely contributes to the ability to generate faster contractions in cells while the higher duty ratio of NM2B suggests that it can bear more load [8].

An important tool that has significantly contributed to our understanding of NM2 function is the popular myosin-2 inhibitor blebbistatin and its derivatives [71, 72]. Blebbistatin is an uncompetitive inhibitor that almost completely inhibits the ATPase activity of all NM2 paralogs with low micromolar affinity (IC_50_ ~ 0.5-5 µM) by trapping myosin in the weak actin binding ADP·P_i_ state, thus slowing down phosphate release and entry into the strong actin binding state [73–75]. Recently, it has been reported that the small molecular inhibitor of formin FH2 domains, SMIFH2, inhibits myosin motors including NM2A with an IC_50_ of ~ 50 µM in vitro and NM2A-dependent cellular functions at lower concentrations in cells [76]. The observed cross-reactivity of SMIFH2 suggests that this inhibitor should be used with extreme caution and rigorous controls when studying either formin or NM2, as it may introduce potential confounding effects.

### Structure

A significant advance in our understanding of the molecular mechanisms of myosin motors occurred when the structure of a single-headed myosin-2 fragment was published ~ 30 years ago [38]. Since then, high-resolution crystal structures of human NM2B (PDB ID: 4PD3, nucleotide-free state) and human NM2C (PDB ID: 5I4E, pre-powerstroke state) motor domains in different nucleotide states as well as a cryo-EM structure of the human NM2C (PDB ID: 5JLH, rigor state) motor domain bound to actin have been solved [61, 77, 78]. Though the basic features of the motor domain are common to all myosins-2 structures solved to date, including those from model organisms, they are valuable resources to understand the allosteric nature of myosin motor function that lead to kinetic fingerprints and paralog- and isoform-specific cellular activity profiles [38, 60].

### Motor domain

The highly conserved mechanochemical core of the NM2 motor domain is composed of a seven-stranded β-sheet flanked by α-helices on each side, reminiscent of that found in other myosins and evolutionarily related G proteins and kinesins (Fig. 2a) [38, 79, 80]. This arrangement generates four functional and allosteric subdomains in the myosin motor domain: The N-terminal subdomain (Nter), the upper 50 kDa subdomain (U50), the lower 50 kDa subdomain (L50), and the converter (Fig. 2a) [60, 81]. The U50 and the L50 are separated by a large cleft in the absence of actin [38].

The nucleotide binding pocket is the catalytic core of NM2 (Fig. 2b). It is formed by structural elements of the U50 and features three conserved nucleotide binding motifs: P-loop (GESGAKT), switch-1 (NXNSSRGF), and switch-2 (DIXGFE) [79]. Nucleotide binding to the motor domain is sensed by the phosphate-binding P-loop and causes the movement of switches-1 and − 2 during the myosin ATPase cycle. Switch-1 and − 2 form a characteristic salt bridge in the closed conformation that is critical for the hydrolysis of ATP [38, 82–84]. The closed conformation of the nucleotide binding pocket is linked to the converter and the lever arm in the ‘up’ position (ATP, ADP·P_i_) while an open nucleotide binding pocket is associated with the converter and the lever arm in the ‘down’ position (apo, ADP). The different converter and lever arm conformations are allosterically communicated with the nucleotide state of the nucleotide binding pocket through conformational changes in the relay helix. The Nter/converter/lever arm interface is critical for NM2 kinetic signatures and its distortion is thought to contribute to the reported load-sensitivity of NM2 paralogs [77].

The ~ 10 Å long neck domain distal to the myosin motor domain functions as a lever arm. It amplifies small-scale movements in the motor domain to a large scale, 60–70˚ movement (Fig. 2c), thereby forming the structural basis for the powerstroke of NM2s [79]. The nucleotide binding pocket is flanked by the JK-loop. A reduction in the length of this loop in NM2C disrupts key interactions with switch-1 and the nucleotide and renders the active site more assessable compared to other myosin-2 paralogs, possibly contributing to their low catalytic efficiency [77].

The actin binding region is a large and dynamic interface that is formed by structural elements of the U50 and the L50. It is located > 40 Å from the nucleotide binding pocket. Changes in the actin binding interface in response to nucleotide binding, hydrolysis, and product release are reflected in differential affinities of myosin for actin [60]. The movement of switch-1 in response to nucleotide binding and release is linked to the opening/closing of the cleft between the U50 and the L50 [83]. The high-resolution cryo-EM structure of NM2C in the strong actin binding apo state revealed that the interface between both proteins is mainly stabilized by hydrophobic interactions [61]. Actin binding is initiated by hydrophobic interactions with loop-2 that connects the L50 and the U50 of the motor domain and plays a central role during the initial formation of the actomyosin interface [61]. This interaction is thought to promote the closure of the cleft that is required for the strong binding of myosin to actin. The helix-loop-helix motif (HLH) in the L50 contributes to a strong actin binding interface through hydrophobic and electrostatic interactions with actin [61]. The CM-loop in the U50 interacts with actin through hydrophobic and weak electrostatic interactions. Loop-3 of the L50 engages in weak charged interactions with actin [61]. The structure also revealed that in NM2C, the activation loop that has been suggested to directly bind to actin and activate the enzyme function of some class-2 myosins, is part of the actomyosin interface in the apo state [61, 85].

### Alternative splicing in the motor domain

Kinetic and functional signatures of NM2s are fine-tuned by alternative splicing in flexible surface loops-1 and − 2 that extend the core of the myosin motor domain. This generates a large number of NM2 isoforms from a small number of genes [5, 86, 87]. NM2 isoforms have either an insert in loop-1 (A1, B1, C1), an insert in loop-2 (A2, B2, C2) or both inserts (B1B2, C1C2) [5, 86, 87]. Loop-1 is located in the U50 and is a key regulator of ADP kinetics. Compared to isoforms with uninserted loops (A0, B0, C0), an extended loop-1 allosterically accelerates the ADP release and therefore the in vitro sliding velocity of NM2s, similar to what has been observed with smooth muscle myosin-2 [51, 55, 88]. NM2 isoforms with extended loops-2 have a higher catalytic activity (k_cat_) under steady-state conditions and increased actin binding rate constants in the presence and absence of ADP [55]. Alternatively spliced loops-1 and − 2 not only fine-tune NM2 kinetics but also affect the regulation of NM2 isoforms in vitro. For example, the NM2C isoform with the C2 insert is constitutively active and does not require RLC phosphorylation for maximum actin-activated steady-state ATPase activity and in vitro motility [89]. This is in striking contrast to the NM2B isoform with the B2 insert that lacks actin-activated steady-state ATPase activity and in vitro motility after RLC phosphorylation [90]. The structural origin of these differences remains unclear since the B2 insert does not interfere with the formation of the autoinhibited (10S) state and/or filament formation, as discussed below, suggesting an alternate mode of regulation that directly affects kinetics. Notably, subtle changes in the kinetic properties are indispensable for the specialized in vivo functions of NM2 isoforms as they have individual expression patterns, intracellular localizations, and binding partners [11, 91–93]. For example, the B1 isoform is expressed during embryonic development whereas the B2 isoform is only expressed after birth [91]. The ablation of the B1 isoform results in the abnormal migration of facial neurons and the development of hydrocephalus in a mouse model [91]. In contrast, the ablation of the B2 isoform results in the abnormal maturation of cerebellar Purkinje cells and impaired motor coordination [91]. The observation that homologous mutations have distinct effects in different NM2 paralogs and isoforms suggests individual allosteric regulation mechanisms [51]. Alternate splicing in surface loops therefore creates kinetic, regulatory, and functional diversity in NM2 paralogs.

### Neck domain and tail domain

The neck domain is a short ~ 10 Å long α-helical structure that features two IQ motifs [94]. The first consensus IQ motif ([I, L,V]QXXXRXXXX[R, K]) is the binding site for the ELC and the second divergent IQ motif is the binding site for the RLC [38, 40]. A sharp bend, the hook, features a conserved WQWW motif that interacts with a portion of the RLC and connects the neck to the tail domain [38]. An invariant proline marks the neck/tail junction and helps in orienting the myosin motor domains to engage with actin.

The tail domain forms a long α-helix and ends in an NHT. The tail domains of two myosin heavy chains dimerize into an extended left-handed coiled-coil. The coiled-coil is formed from a series of consensus heptad repeats *(a-b-c-d-e-f-g)*_*n*_ in which positions *a* and *d* are commonly occupied by hydrophobic amino acids [95, 96]. A 28 amino acid repeat pattern of four heptads along the length of the tail produces an alternating pattern of positive and negative charged zones that have been implicated in the parallel and antiparallel alignment of adjacent tail domains in an NM2 filament [97–99]. The periodic 28 amino acid repeat is interrupted at 3 places by the insertion of skip residues that introduce regions of flexibility in the coiled-coil and lead to local structural changes and discontinuities [100, 101]. The exact role of the individual skip residues in NM2s has yet to be explored but they are likely to keep the alternating pattern of positive and negative charged zones in phase to promote proper filament assembly, as it has been shown for muscle myosin-2s [100, 102].

The end of the tail domain contains two ~ 35–39 amino acid long assembly competence domains (ACD) and ends in a ~ 35–43 amino acid long NHT [103–105]. The two conserved ACDs, ACD1 and ACD2, of two NM2 monomers form an antiparallel homodimer through electrostatic interactions [103, 106, 107]. The high sequence conservation in the ACD between the three NM2 paralogs allows them to assemble into either homotypic filaments but also into heterotypic and mixed filaments [106]. While homotypic filaments are composed of one NM2 paralog, heterotypic filaments are composed of two NM2 paralogs, whereas mixed filaments are composed of a NM2 paralog and a small number of enzymatically inactive myosin-18A molecules [42, 43, 46]. The NHT is absent in sarcomeric myosins, suggesting that its presence in NM2s and smooth muscle myosin-2 is connected with the ability of the latter myosins to dynamically assemble and disassemble to meet contractile cellular demands [104]. The exact contribution of the NHT on the in vitro assembly of NM2s has not been systematically addressed with full-length proteins so far and deletion studies with NM2 tail fragments, which tend to form non-physiological paracrystals opposed to discrete bipolar filaments, have resulted in conflicting results [104, 108, 109]. The presence of phosphorylation sites within the coiled-coil tail domain and the NHT further suggest regulatory functions, as discussed below [110]. In cells, filament assembly, and disassembly are likely modulated by the synergistic effect of the NHT and tail phosphorylation [111]. While the aforementioned domains and structural motifs are conserved across mammalian NM2s, the NM2B heavy chain additionally features a ~ 7 amino acid long regulatory motif (RM) between the coiled-coil tail and the NHT [112]. The RM contains five serines out of which several are phosphorylated in cells. Phosphorylation of S1935 has been identified as the primary regulatory site that controls NM2B assembly and dynamics during front-back polarization in migrating cells [112].

### Filaments and higher-order organization

NM2 exists in two global states: autoinhibited (10S) and filamentous. In the presence of ATP and the absence of phosphorylation of S19 in the RLC (S19_RLC_), NM2 assumes a compact, autoinhibited conformation known as 10S in reference to its sedimentation coefficient [113, 114]. Myosin in this conformation is thought to be diffusible and assembly-incompetent [10]. In the 10S conformation, the two motor domains, the blocked head (BH) and the free head (FH), asymmetrically dock against each other in a conformation known as the interacting heads motif (IHM) in analogy to the structure that likely contributes to the super-relaxed state of cardiac muscle myosin-2 [115, 116]. The tail folds in three places, loops around the IHM, and folds back on itself [117]. This complex folding of the monomer inhibits the release of P_i_ ~ 100-fold and traps the enzymatically inactive myosin in a weak actin-binding ADP·P_i_ state (K_D_ > 100 µM) [118]. Phosphorylation of S19_RLC_ relieves the 10S state and promotes filament formation, as discussed below [114, 119, 120]. The dynamic shape changes from the 10S to the filamentous state and vice versa are important to drive biological processes associated with the mechanical properties of cells [10, 121, 122]. The molecular mechanism and structural changes that drive the activation mechanism have recently been revealed by high-resolution cryo-EM of the closely related smooth muscle myosin-2 [123–125]. The structure showed that in 10S, the RLC_BH_ and RLC_FH_ adopt unique conformations that bury the phosphorylatable S19 of the RLC_FH_ [123]. The phosphorylatable S19_RLC_ on the RLC_FH_ contacts the folded tail and is accessible for kinases [123]. This suggests that 10S relief by phosphorylation is a sequential process that involves the phosphorylation of S19_RLC_ on the RLC_FH_, a series of conformational changes that weaken the RLC_BH_:RLC_FH_ interface and finally make S19 on the RLC_BH_ available for phosphorylation by RLC kinases [123]. In addition, the pathway likely involves changes in the interactions between the RLC and the ELC as well as the ELC and the motor domain [123]. Importantly, S19_RLC_ phosphorylation does not directly activate the enzymatic activity of NM2 but rather prevents myosin from adopting the IHM which shuts down the activity. Evidence in support of this included the observation that single-headed NM2 fragments from higher eukaryotes are constitutively active and not regulated by phosphorylation [55–57, 62, 69]. Upon RLC phosphorylation, NM2 monomers assemble into bipolar, highly organized filaments through a well-defined axial staggering of their coiled-coil tail domains via electrostatic interactions, as described above [98, 103].

**Fig. 3:**
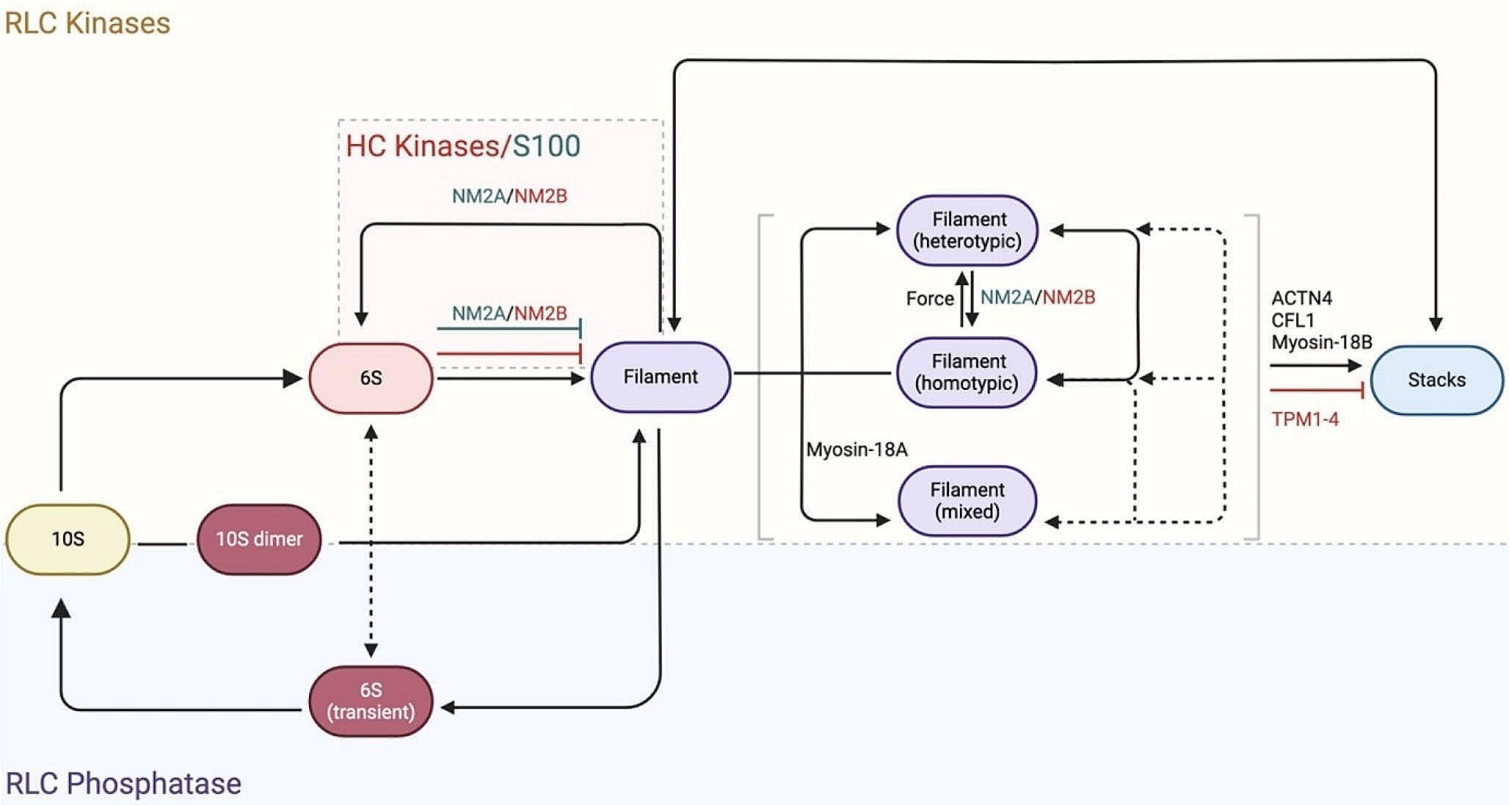
Regulation mechanisms of NM2. Select regulation mechanisms of NM2s reviewed in this work. Grey and red font colors indicate regulation mechanisms specific for NM2A and NM2B, respectively. Dashed lines indicate putative regulation mechanisms. Force: force dependent maturation of heterotypic filaments

Self-assembly and force generation are tightly coupled and essential for the biological function of NM2 paralogs. The structural and molecular mechanisms that underlie the processes of filament assembly and disassembly however are less well understood. Negative staining electron microscopy and computational studies suggest that NM2s assemble from 10S, antiparallel 10S dimers, and possibly tetramers and higher order oligomer intermediates that unfold and associate with an existing filament through rolling and zipping motions (Fig. 3) [126, 127]. However, quantitative analytical ultracentrifugation and interferometric scattering measurements showed that the 10S state is predominant under physiological conditions and only a very small portion of myosin molecules may form transient higher-order oligomers [23, 113], indicating that our understanding of the processes of filament assembly as well as disassembly is still incomplete.

In a filament, ~ 29 (NM2A) or ~ 30 (NM2B) monomers work cooperatively to produce force and motion in the actin cytoskeleton. NM2C filaments are thinner and contain ~ 14 monomers [117]. NM2 filaments have a contour length of ~ 300 nm, a width between ~ 7.9–11.5 nm, and bare zones between ~ 166–219 nm [117, 128, 129]. Due to the significantly shorter length and the reduced number of heavy chains compared to striated muscle myosin-2 filaments, NM2 filaments are also referred to as minifilaments. A mature minifilament composed of 30 monomers has five crowns of three monomers that are projecting out on each side based on *in silico* studies [126]. The RLC-phosphorylated motor domains are splayed away from the filament backbone, allowing them to survey for actin binding sites within tens of nanometers [117, 126]. Negative staining electron microscopy showed that NM2 filaments can bind single or multiple actin filaments either via one or both ends of the myosin filament [117, 130]. This likely determines the interaction and force generation of NM2s on actin structures with different nanoarchitectures in the cell that can give rise to contractile and potentially extensile forces [131]. Interestingly, a recent super-resolution microscopy study showed that in the cell cortex of interphase cells, NM2 minifilaments do not fully overlap with actin. A reduced actin-myosin overlap has been correlated with reduced cortical tension, leading to the speculation that minifilaments may only be bound to actin filaments with one end in cells, as it has been observed in vitro [9, 117, 130].

NM2 paralogs can not only assemble into homotypic filaments but also into heterotypic and mixed filaments (Fig. 3) [42, 43, 46]. The co-assembly of myosins at different ratios gives rise to filaments with a broad range of kinetic and dynamic properties that are needed to perform specialized functions during processes including cell migration and division [132, 133]. For example, the co-assembly of NM2A into an NM2B filament is expected to decrease the collective duty ratio and accelerate filament dynamics and biochemical properties. Likewise, in vitro experiments showed that the incorporation of NM2B into a non-processive NM2A filament allows the heterotypic filament to move processively on actin filaments [65]. This also suggests that the slower myosin determines the speed of movement of the heterotypic filament [65]. *In silico* studies revealed that homotypic filaments of NM2A and heterotypic NM2A/NM2B filaments are stable, whereas NM2B and NM2C filaments appear less stable [126]. Mathematical modeling further proposed that the ATPase activity of NM2 paralogs directly translates into the observed differences in minifilament stability [134].

A mechanistic model suggests that if NM2 paralogs co-assemble with equal probability and proportionally to their cellular availability, a gradient distribution of NM2A, mixed NM2A/B, NM2B/A, and NM2B would emerge from the front to the rear in migrating cells [132]. This spatial distribution of minifilaments has been experimentally observed in cells [121, 132, 134–136]. In knock-out cell lines, it was shown that NM2A and NM2B play a distinct role during filament formation. Quantitative imaging and mathematical modeling suggest that NM2A forms initiating filaments to which NM2B incorporates. In those heterotypic filaments, NM2B acts as a break to limit the collective activity of filaments containing NM2A [134]. Further, it has been reported that the actin mesh size limits the degree of penetration of myosin minifilaments into the cell cortex by steric effects, suggesting that the degree of penetration determines cortical tension [9]. Notably, NM2A and NM2B localize to nearly identical structures such as cell-cell junctions and large contractile bundles while NM2C is absent from stress fibers and larger contractile bundles in epithelial cells [137, 138]. It was also shown that NM2C localizes independently from NM2A in brush border microvilli [139].

NM2 filaments are dynamic assemblies that split, concatenate, and form higher-order stacks in cells [44, 122, 140]. In stacks, dozens of parallel NM2 filaments stack on one another and form a sarcomere-like registry perpendicular to actin bundles [44, 128, 141]. Stacks have been observed in various cell types and appear to be involved in the positioning and movement of actin bundles and the regulation of contractile actomyosin structures for example in interphase and dividing cells [44, 45, 122, 140, 142]. In stress fibers, myosin-18B localizes at the ends of NM2 stacks and contributes to the assembly of large stacks that are critical for the generation of contractile actomyosin bundles [45]. High-resolution imaging further revealed that stacks are associated with the minus end of the actin filament [44]. Stacks can span several actin bundles that are separated by ~ 100 nm, suggesting long-range interactions. Stacks are formed by the parallel fusion of adjacent individual filaments and small stacks into large stacks and disassemble by splitting [44, 45, 122, 140, 143]. Stack formation is a slow process that takes tens of minutes and depends on NM2 motor activity and the balanced activities of actin binding (ABP), crosslinking, and regulatory proteins [44, 45, 122]. The ABPs α-actinin-4 and cofilin1 in addition to the actin assembly factor formin-like 3 and unconventional myosin-18B are positive regulators of NM2 stack formation whereas tropomyosin isoforms 1–4 are negative regulators (Fig. 3) [44, 45, 47, 144]. Recent studies further suggested that in addition to NM2 filament assembly and disassembly, cells can also regulate the degree of order in their long-range organization which is important to match their force requirement [144].

NM2 stacks have also been observed at cell-cell junctions [142, 145]. In contrast to stacks observed in fibroblasts, NM2 is organized in extensive, micrometer-scale arrays of stacks in adherens junctions of epithelial cells [145]. Extensive arrays of stacks that span ~ 4–6 μm are formed in locations of more loosely organized actin networks in younger junctions. During junction maturation, the span of these arrays narrows to ~ 200–300 nm without a significant change in the spacing between individual stacks [145]. Stack narrowing likely contributes to the bundling of actin filaments and the formation of sarcomere-like actomyosin arrays at mature junctions, suggesting that the self-organization of NM2 contributes to the organization of the actin cytoskeleton [145].

Biochemical studies on stacks significantly lagged behind cellular studies. Hence, the molecular mechanisms and structural basis of stacks are largely unexplored. Negative staining electron microscopy suggested that stacks are formed by lateral and serial interactions of single NM2 filaments [65, 117]. Since the merging of individual filaments into stacks and the splitting of stacks has been observed in a 2-dimensional in vitro motility assay, it has been proposed that the electrostatic forces that hold stacks together are of similar magnitude as the force exerted by a myosin filament on actin [65]. Interestingly, disease-causing mutations in both the NM2 motor and the tail domain have been shown to alter stack formation in cells without affecting the capability of myosin molecules to assemble into filaments in vitro per se [19, 50]. However, filament parameters including width, length, number of NM2 molecules per filament, and the length of the bare zone show mutant-specific changes, suggesting a complex effect of mutations on filament and stack dimensions [50].

### Regulation mechanisms

NM2 activity is tightly controlled at multiple levels by synergistic regulatory mechanisms that work in concert to ensure *bona fide* cellular function. Factors that influence NM2 function in cells include for example the complex intracellular environment, compartmentalization, protein-protein interactions, posttranslational modifications, and mechanical forces.

### Regulatory light chain phosphorylation

The reversible phosphorylation of the RLC by opposing activities of numerous kinases and myosin light chain phosphatase (MLCP) is tightly controlled by many signaling pathways to regulate NM2 dynamics and assembly (Fig. 3) [119, 146–148]. Super-resolution microscopy studies revealed that the two major RLC kinases – myosin light chain kinase (MLCK) and Rho-associated protein kinase (ROCK) - compete for a limiting pool of NM2 monomers for the spatiotemporal control of actomyosin contractility in cells [122]. Biochemical studies revealed that phosphorylation of the S19_RLC_ promotes autoinhibition relief, filament formation, and therefore the spatial and temporal control of contractility and force generation, as reviewed in greater detail elsewhere [3, 6, 30, 48]. Phosphorylation of the RLC on T18 (T18_RLC_) – in addition to S19 – has a small effect on the enzyme activity of NM2 but T18_RLC_/S19_RLC_-phosphorylated myosin differentially regulates adhesion and polarity in migrating cells and the assembly and stability of NM2 paralogs [149, 150]. At the mitotic cortex, it was shown that MLCK and ROCK preferentially create short-lived S19_RLC_-phosphorylated and long-lived T18_RLC_/S19_RLC_-phosphorylated NM2 species, respectively, suggesting a graded effect on NM2 turnover [151]. While phosphorylation of residues S1, S2, and T9 by protein kinase C (PKC) has been reported to increase the K_app_ of myosin light chain kinase for the RLC in vitro, the physiological significance of this inhibitory phosphorylation remains to be established [152–154]. MLCP dephosphorylates serine and threonine residues on the RLC [155, 156]. Phosphatase activity is inhibited through phosphorylation of its regulatory myosin binding subunit (MYPT1) by ROCK and other kinases [155–158]. Therefore, the dual action of some kinases to promote RLC phosphorylation determines the extent, duration, and location of actomyosin contractility which is counterbalanced by MLCP. It is of note that a recent super-resolution microscopy study reported that the level of S19_RLC_ phosphorylation scales with the expression level of *MYH9* and to a lower degree with the expression of *MYH10* while the expression of *MYH14* does not scale with S19_RLC_ phosphorylation [134]. Together with the observation that the loss of *MYH9* expression negatively affects the assembly of NM2B, this suggests that the local activation of NM2A through S19_RLC_ phosphorylation and the subsequent assembly into filaments likely facilitates the co-assembly of NM2B into existing NM2A filaments [134].

In addition to the phosphorylation of serine and threonine residues, tyrosine phosphorylation of the RLC has been investigated [147, 159]. Phosphorylation of Y155 by epidermal growth factor receptor interferes with the interaction between the RLC and the NM2 heavy chain and filament assembly [147]. Y155 phosphorylation is spatially restricted to the lamellipodium and contributes to the compartmentalization of NM2 assembly in cells [147].

### Heavy chain phosphorylation

While light chain phosphorylation is a paralog-redundant mechanism of NM2 regulation, myosin heavy chain phosphorylation is predominantly paralog-specific (Fig. 3). Unlike in lower eukaryotic myosin-2s, designated myosin heavy chain kinases have not been identified in mammalian cells where several common kinases including PKC isoforms, casein kinase 2 (CK2), and p21-activated kinase 1 (PAK1) have been shown to phosphorylate serine and threonine residues in the NM2 tail [160–163]. Tyrosine phosphorylation in the myosin heavy chain has also been reported but the physiological consequences remain to be explored [164, 165]. Sites and consequences of phosphorylation events in the tail are largely paralog-specific. In some but not all cases, phosphorylation has been shown to modulate the delicate equilibrium between the myosin monomer and filament pool in cells [15, 50, 112, 160]. For example, tail phosphorylation by PKCξ has been shown to control the assembly state of NM2B and therefore the ability to mechanorespond in cells [15]. In vitro, it was shown that CK2 phosphorylation or phosphomimetic mutations at PKC and TRPM7 target sites are insufficient to completely disassemble NM2B tail fragments. Based on this observation, a synergistic effect was proposed in that the accumulation of negative charges through multiple phosphorylation events may weaken the electrostatic interactions between tails in favor of a disassembly pathway for NM2B [161]. Interestingly, the effect of phosphorylation on filament stability has been shown to be more pronounced for NM2B than NM2A, leading to the speculation that tail phosphorylation may selectively fine-tune the composition of NM2A/NM2B heterotypic filaments [161]. As for studies with NHT deletion mutants, the literature on the biochemistry and consequences of tail phosphorylation often reports conflicting results that are not directly supported by cell biological data. To some extent, this may be attributed to the use of recombinant tail fragments of different lengths that may not represent useful models for full-length myosin, phosphomimetic mutations that often insufficiently mimic the phosphorylated state of the protein, the lack of complex phosphorylation patterns produced by multiple kinases and/or the absence of regulatory proteins, suggesting that our understanding of the effect of tail phosphorylation is still incomplete [161, 166–168].

### Interacting partners

Interacting partners can directly and/or indirectly influence myosin activity. They either directly influence the enzyme activity of myosin or interact with the myosin heavy chain. The prime regulator of the enzymatic and functional activity of myosin is filamentous actin, as discussed above. While all actin isoforms stimulate the enzymatic activity of NM2, the extent differs up to ~ 4-fold for NM2A and NM2B [169]. Both myosins have increased ATPase activities, coupling efficiencies and in vitro gliding speeds with β- and γ-actin over α-actin [169]. NM2C is most potently activated by β-actin [169]. Recent cryo-EM structures suggest that the divergent N-termini of actin isoforms adopt distinct conformations that may be sensed by and guide the binding of NM2 paralogs [170]. The resulting distinct actomyosin interfaces are likely to contribute to the reported enzymatic and functional differences [170]. Posttranslational modifications of the N-termini of actin isoforms such as acetylation and arginylation are predicted to further modulate the actin-NM2 interface [170]. Other posttranslational modifications such as ADP-ribosylation of α-actin on T148 by the bacterial toxin TccC3 have no significant effect on the steady-state kinetics of NM2B [171]. In cells, the majority of actin filaments are associated with tropomyosins as well as other ABPs. Decoration of actin with tropomyosin isoforms showed varying effects on gliding speeds and ATPase activities of NM2 paralogs in vitro, suggesting that the paralog itself is the major determinant of the observed effects on the actin-myosin interaction [172–175]. Further, N-terminal acetylation of tropomyosin impacts the actin-activated ATPase activity and in vitro motility of NM2A in an isoform-specific manner [175].

Modulation of the basic actin-myosin interaction by alternatively spliced surface loops in NM2, several posttranslational modifications of actin isoforms and ABPs that diversify actin cytoskeletal structure, generates countless actin-ABP-NM2 combinations that may modulate cytoskeletal function in time and space. This suggests that the local composition of the actin cytoskeleton fine-tunes the enzymatic output of NM2 motors which may also contribute to myosin sorting and the generation of different actin landscapes in cells.

The myosin tail domain is also a binding site for interacting partners that negatively affect NM2 filament assembly and/or disassembly and therefore may change the equilibrium between the homotypic and the heterotypic filament pool and paralog sorting [132, 161]. Negative regulators of NM2A filament assembly include structurally diverse proteins including myosin binding protein H, lethal giant larvae, and members of the S100 protein family [176–178]. The latter selectively disassemble NM2A filaments and/or remove NM2A from heterotypic filaments and have only very little effect on NM2B, where tail phosphorylation seems to be the predominant regulator of filament assembly and disassembly [161, 178, 179]. The selective removal of RLC-phosphorylated NM2A from filaments through S100A family proteins would likely result in enzymatically activated 6S monomers [46].

Structural studies on complexes between Ca^2+^-S100A4 and NM2A tail peptides revealed a binding interface at the junction of ACD1 and the NHT [106, 179, 180]. The structural information led to the model that S100A4 binds to the NHT to initiate partial unzipping of the coiled-coil which may subsequently destabilize the staggered packing of myosin molecules in the filament and finally result in filament dissociation (Fig. 3) [179, 180]. The structural basis for this model is based on the observation that the NHT and the C-terminus of the ACD1 wrap around a Ca^2+^-loaded S100A4, thereby disrupting the ACD and promoting filament disassembly [179]. For other NM2A binding partners such as the Arf-GTPase-activating protein ASAP1 and cingulin, a possible effect on filament assembly and disassembly is still being defined [181–183].

### Mechanical forces

Piconewton forces guide and direct the behavior of cells [184]. As mechanoenzymes, NM2s not only generate forces by pulling on antiparallel actin filaments but also sense and transmit forces and/or alter the actin cytoskeleton in complex ways. Thereby, NM2s directly and indirectly promote and influence processes including cell mechanics and contractility, mechanosensing, cellular elasticity, cortical tension, and adhesion mechanisms [9, 185–188].

It has been shown that NM2s are mechanosensitive proteins that sense and respond to chemical and mechanical forces by dynamically changing their enzymatic output in vitro and in cells [59, 63, 174, 188–191]. This in turn may be sensed by other proteins and lead to coherent cellular responses for example during focal adhesion maturation [186, 187]. The force-sensitivity of NM2B also contributes to increased tension generation and actin-crosslinking during cytokinesis that resists the expansion of the ring [192].

The altered kinetic pathways that underlie the force-sensitivity of NM2 paralogs are not fully defined yet. In vitro kinetic studies showed that the actin-activated ADP release increases with assisting loads and decreases with resisting loads [189, 193]. The effect of resisting loads on the ADP release is more pronounced for NM2B (12-fold) compared to NM2A (5-fold). Resisting loads increase the duty ratio and result in long actin attachment lifetimes that may reach into the minute timescale to favor tension maintenance without hydrolyzing ATP [189]. Thus, actomyosin interactions can be classified as either catch- or slip-bonds depending upon whether the actin-attachment lifetime increases or decreases with force [97, 188]. Catch-bond behavior is likely to underlie the force-dependent accumulation of NM2 to stressed parts of the actin cytoskeleton independent of the cellular signaling [15, 190, 191, 194]. While it remains controversial whether a mammalian NM2 monomer can move processively, optical trapping studies suggest that the detachment rate of NM2A and NM2B from filamentous actin is reduced under resisting load that slows down the rate of ADP release [66, 174]. Tropomyosin 4.2-decorated actin filaments have been shown to amplify this effect in vitro with possible implications for the physiological role of this myosin to stabilize specialized cellular actin structures such as stress fibers [174]. It is also likely that the force-sensitivity of NM2 paralogs contributes to the ‘latch’ state in tonic smooth muscle, during which force is maintained for prolonged periods at low rates of ATP consumption [195].

While the precise structural mechanisms for the described force sensitivity have not been fully resolved, it has been proposed that NM2s undergo force-dependent conformational changes [196]. Negative stain electron microscopy demonstrated that binding of myosin with both motor domains to neighboring sites on an actin filament generates sufficient internal strain to drive load-depending kinetics [189]. In addition, structural studies revealed an allosteric communication pathway that connects the active site with the distal end of the motor domain. Mutagenesis experiments suggested that distortion of the converter/lever arm interface under strain may disrupt this pathway and contribute to the reported load-dependent kinetics of NM2s [77].

Together, the mechanokinetic tuning of myosin enzymology and assembly through forces represents an additional layer of NM2 regulation matched to their physiological environment.

### Concluding remarks

Recent advances in the field have answered many long-standing questions and revealed an unexpected new complexity of NM2 function and regulation. Remaining and new questions include the existence and cellular roles of the activated 6S monomer, how the tail coiled-coils wind and unwind to assemble or disassemble filaments, the visualization of 10S in cells and probes for the 10S–6S transition, the exact mechanism of NM2 filament formation, and the roles and ultrastructure of NM2 stacks will be addressed with novel technologies in the future to deepen our understanding of the fundamental mechanisms of myosin motor function and to facilitate the development of new research tools and therapeutic strategies for myosin-related diseases. The integration of biochemistry, structural biology, physics, advanced light and electron microscopy will ultimately lead to a more comprehensive and quantitative understanding of NM2 function underlying biological processes and mechanisms in health and disease and provide exciting opportunities for discovery and innovation.

## Data Availability

Not applicable.

## References

[1] Lee LA, Karabina A, Broadwell LJ, Leinwand LA (2019). The ancient sarcomeric myosins found in specialized muscles. Skelet Muscle.

[2] Odronitz F, Kollmar M (2007). Drawing the tree of eukaryotic life based on the analysis of 2,269 manually annotated myosins from 328 species. Genome Biol.

[3] Vicente-Manzanares M, Ma X, Adelstein RS, Horwitz AR (2009). Non-muscle myosin II takes centre stage in cell adhesion and migration. Nat Rev Mol Cell Biol.

[4] Ma X, Adelstein RS (2014). The role of vertebrate nonmuscle myosin II in development and human disease. Bioarchitecture.

[5] Golomb E, Ma X, Jana SS, Preston YA, Kawamoto S, Shoham NG, Goldin E, Conti MA, Sellers JR, Adelstein RS (2004). Identification and characterization of nonmuscle myosin II-C, a new member of the myosin II family. J Biol Chem.

[6] Sellers JR, Heissler SM (2019). Nonmuscle myosin-2 isoforms. Curr Biol.

[7] Kothari P, Johnson C, Sandone C, Iglesias PA, Robinson DN (2019) How the mechanobiome drives cell behavior, viewed through the lens of control theory. J Cell Sci 132. 10.1242/jcs.234476 10.1242/jcs.234476 PMC6771144 31477578

[8] Weissenbruch K, Grewe J, Hippler M, Fladung M, Tremmel M, Stricker K, Schwarz US, Bastmeyer M (2021) Distinct roles of nonmuscle myosin II isoforms for establishing tension and elasticity during cell morphodynamics. Elife 10. 10.7554/eLife.71888 10.7554/eLife.71888 PMC8391736 34374341

[9] Truong Quang BA, Peters R, Cassani DAD, Chugh P, Clark AG, Agnew M, Charras G, Paluch EK (2021). Extent of myosin penetration within the actin cortex regulates cell surface mechanics. Nat Commun.

[10] Baird MA, Billington N, Wang A, Adelstein RS, Sellers JR, Fischer RS, Waterman CM (2017). Local pulsatile contractions are an intrinsic property of the myosin 2A motor in the cortical cytoskeleton of adherent cells. Mol Biol Cell.

[11] Wang A, Ma X, Conti MA, Liu C, Kawamoto S, Adelstein RS (2010). Nonmuscle myosin II isoform and domain specificity during early mouse development. Proc Natl Acad Sci U S A.

[12] Ma X, Bao J, Adelstein RS (2007). Loss of cell adhesion causes hydrocephalus in nonmuscle myosin II-B-ablated and mutated mice. Mol Biol Cell.

[13] Ma X, Adelstein RS (2012). In vivo studies on nonmuscle myosin II expression and function in heart development. Front Biosci (Landmark Ed.

[14] Conti MA, Even-Ram S, Liu C, Yamada KM, Adelstein RS (2004). Defects in cell adhesion and the visceral endoderm following ablation of nonmuscle myosin heavy chain II-A in mice. J Biol Chem.

[15] Schiffhauer ES, Ren Y, Iglesias VA, Kothari P, Iglesias PA, Robinson DN (2019). Myosin IIB assembly state determines its mechanosensitive dynamics. J Cell Biol.

[16] Ma X, Jana SS, Conti MA, Kawamoto S, Claycomb WC, Adelstein RS (2010). Ablation of nonmuscle myosin II-B and II-C reveals a role for nonmuscle myosin II in cardiac myocyte karyokinesis. Mol Biol Cell.

[17] Kage F, Vicente-Manzanares M, McEwan BC, Kettenbach AN, Higgs HN (2022). Myosin II proteins are required for organization of calcium-induced actin networks upstream of mitochondrial division. Mol Biol Cell.

[18] Parajon E, Surcel A, Robinson DN (2021). The mechanobiome: a goldmine for cancer therapeutics. Am J Physiol Cell Physiol.

[19] Sung DC, Ahmad M, Lerma Cervantes CB, Zhang Y, Adelstein RS, Ma X (2021). Mutations in non-muscle myosin 2A disrupt the actomyosin cytoskeleton in sertoli cells and cause male infertility. Dev Biol.

[20] Pecci A, Ma X, Savoia A, Adelstein RS (2018). MYH9: structure, functions and role of non-muscle myosin IIA in human disease. Gene.

[21] Ivkovic S, Beadle C, Noticewala S, Massey SC, Swanson KR, Toro LN, Bresnick AR, Canoll P, Rosenfeld SS (2012). Direct inhibition of myosin II effectively blocks glioma invasion in the presence of multiple motogens. Mol Biol Cell.

[22] Picariello HS, Kenchappa RS, Rai V, Crish JF, Dovas A, Pogoda K, McMahon M, Bell ES, Chandrasekharan U, Luu A, West R, Lammerding J, Canoll P, Odde DJ, Janmey PA, Egelhoff T, Rosenfeld SS (2019). Myosin IIA suppresses glioblastoma development in a mechanically sensitive manner. Proc Natl Acad Sci U S A.

[23] Young EJ, Blouin AM, Briggs SB, Sillivan SE, Lin L, Cameron MD, Rumbaugh G, Miller CA (2016). Nonmuscle myosin IIB as a therapeutic target for the prevention of relapse to methamphetamine use. Mol Psychiatry.

[24] Tullio AN, Accili D, Ferrans VJ, Yu ZX, Takeda K, Grinberg A, Westphal H, Preston YA, Adelstein RS (1997). Nonmuscle myosin II-B is required for normal development of the mouse heart. Proc Natl Acad Sci U S A.

[25] Tullio AN, Bridgman PC, Tresser NJ, Chan CC, Conti MA, Adelstein RS, Hara Y (2001). Structural abnormalities develop in the brain after ablation of the gene encoding nonmuscle myosin II-B heavy chain. J Comp Neurol.

[26] Takeda K, Kishi H, Ma X, Yu ZX, Adelstein RS (2003). Ablation and mutation of nonmuscle myosin heavy chain II-B results in a defect in cardiac myocyte cytokinesis. Circ Res.

[27] Bao J, Jana SS, Adelstein RS (2005). Vertebrate nonmuscle myosin II isoforms rescue small interfering RNA-induced defects in COS-7 cell cytokinesis. J Biol Chem.

[28] Quintanilla MA, Hammer JA, Beach JR (2023) Non-muscle myosin 2 at a glance. J Cell Sci 136. 10.1242/jcs.260890 10.1242/jcs.260890 PMC10411949 36917212

[29] Yamada KM, Sixt M (2019). Mechanisms of 3D cell migration. Nat Rev Mol Cell Biol.

[30] Heissler SM, Manstein DJ (2013). Nonmuscle myosin-2: mix and match. Cell Mol Life Sci.

[31] Amberger JS, Bocchini CA, Schiettecatte F, Scott AF, Hamosh A (2015). OMIM.org: online mendelian inheritance in man (OMIM(R)), an online catalog of human genes and genetic disorders. Nucleic Acids Res.

[32] Lalwani AK, Goldstein JA, Kelley MJ, Luxford W, Castelein CM, Mhatre AN (2000). Human nonsyndromic hereditary deafness DFNA17 is due to a mutation in nonmuscle myosin MYH9. Am J Hum Genet.

[33] Zhang Y, Conti MA, Malide D, Dong F, Wang A, Shmist YA, Liu C, Zerfas P, Daniels MP, Chan CC, Kozin E, Kachar B, Kelley MJ, Kopp JB, Adelstein RS (2012). Mouse models of MYH9-related disease: mutations in nonmuscle myosin II-A. Blood.

[34] Holtz AM, VanCoillie R, Vansickle EA, Carere DA, Withrow K, Torti E, Juusola J, Millan F, Person R, Guillen Sacoto MJ, Si Y, Wentzensen IM, Pugh J, Vasileiou G, Rieger M, Reis A, Argilli E, Sherr EH, Aldinger KA, Dobyns WB, Brunet T, Hoefele J, Wagner M, Haber B, Kotzaeridou U, Keren B, Heron D, Mignot C, Heide S, Courtin T, Buratti J, Murugasen S, Donald KA, O’Heir E, Moody S, Kim KH, Burton BK, Yoon G, Campo MD, Masser-Frye D, Kozenko M, Parkinson C, Sell SL, Gordon PL, Prokop JW, Karaa A, Bupp C, Raby BA (2022). Heterozygous variants in MYH10 associated with neurodevelopmental disorders and congenital anomalies with evidence for primary cilia-dependent defects in hedgehog signaling. Genet Med.

[35] Tuzovic L, Yu L, Zeng W, Li X, Lu H, Lu HM, Gonzalez KD, Chung WK (2013). A human de novo mutation in MYH10 phenocopies the loss of function mutation in mice. Rare Dis.

[36] Lowey S, Cohen C (1962). Studies on the structure of myosin. J Mol Biol.

[37] Lowey S, Slayter HS, Weeds AG, Baker H (1969). Substructure of the myosin molecule. I. Subfragments of myosin by enzymic degradation. J Mol Biol.

[38] Rayment I, Rypniewski WR, Schmidt-Base K, Smith R, Tomchick DR, Benning MM, Winkelmann DA, Wesenberg G, Holden HM (1993). Three-dimensional structure of myosin subfragment-1: a molecular motor. Science.

[39] Weeds AG, Lowey S (1971). Substructure of the myosin molecule. II. The light chains of myosin. J Mol Biol.

[40] Heissler SM, Sellers JR (2014) Myosin light chains: teaching old dogs new tricks. Bioarchitecture 4169–188. 10.1080/19490992.2015.1054092 10.1080/19490992.2015.1054092 PMC4914028 26155737

[41] Nayak A, Wang T, Franz P, Steffen W, Chizhov I, Tsiavaliaris G, Amrute-Nayak M (2020). Single-molecule analysis reveals that regulatory light chains fine-tune skeletal myosin II function. J Biol Chem.

[42] Beach JR, Shao L, Remmert K, Li D, Betzig E, Hammer JA (2015). Nonmuscle myosin II isoforms Coassemble in living cells. Curr Biol.

[43] Billington N, Beach JR, Heissler SM, Remmert K, Guzik-Lendrum S, Nagy A, Takagi Y, Shao L, Li D, Yang Y, Zhang Y, Barzik M, Betzig E, Hammer JA, Sellers JR (2015). Myosin 18A coassembles with nonmuscle myosin 2 to form mixed bipolar filaments. Curr Biol.

[44] Hu S, Dasbiswas K, Guo Z, Tee YH, Thiagarajan V, Hersen P, Chew TL, Safran SA, Zaidel-Bar R, Bershadsky AD (2017). Long-range self-organization of cytoskeletal myosin II filament stacks. Nat Cell Biol.

[45] Jiu Y, Kumari R, Fenix AM, Schaible N, Liu X, Varjosalo M, Krishnan R, Burnette DT, Lappalainen P (2019). Myosin-18B promotes the assembly of myosin II stacks for maturation of contractile actomyosin bundles. Curr Biol.

[46] Shutova MS, Spessott WA, Giraudo CG, Svitkina T (2014). Endogenous species of mammalian nonmuscle myosin IIA and IIB include activated monomers and heteropolymers. Curr Biol.

[47] Latham SL, Weiss N, Schwanke K, Thiel C, Croucher DR, Zweigerdt R, Manstein DJ, Taft MH (2020). Myosin-18B regulates higher-Order Organization of the Cardiac Sarcomere through Thin Filament Cross-linking and Thick Filament dynamics. Cell Rep.

[48] Asensio-Juarez G, Llorente-Gonzalez C, Vicente-Manzanares M (2020) Linking the Landscape of MYH9-Related diseases to the Molecular mechanisms that Control Non-muscle myosin II-A function in cells. Cells 9. 10.3390/cells9061458 10.3390/cells9061458 PMC7348894 32545517

[49] Holtz AM, Vancoil R, Vansickle EA, Carere DA, Withrow K, Torti E, Juusola J, Millan F, Person R, Guillen Sacoto MJ, Si Y, Wentzensen IM, Pugh J, Vasileiou G, Rieger M, Reis A, Argilli E, Sherr EH, Aldinger KA, Dobyns WB, Brunet T, Hoefele J, Wagner M, Haber B, Kotzaeridou U, Keren B, Heron D, Mignot C, Heide C, Courtin T, Buratti J, Murugasen S, Donald KA, O’Heir E, Moody S, Kim KH, Burton BK, Yoon G, Campo MD, Masser-Frye D, Kozenko M, Parkinson C, Sell SL, Gordon PL, Prokop JW, Karaa A, Bupp C, Raby BA (2022). Heterozygous variants in MYH10 associated with neurodevelopmental disorders and congenital anomalies with evidence for primary cilia-dependent defects in hedgehog signaling. Genet Med.

[50] Pal K, Nowak R, Billington N, Liu R, Ghosh A, Sellers JR, Fowler VM (2020). Megakaryocyte migration defects due to nonmuscle myosin IIA mutations underlie thrombocytopenia in MYH9-related disease. Blood.

[51] Kim KY, Kovacs M, Kawamoto S, Sellers JR, Adelstein RS (2005). Disease-associated mutations and alternative splicing alter the enzymatic and motile activity of nonmuscle myosins II-B and II-C. J Biol Chem.

[52] Sitbon YH, Yadav S, Kazmierczak K, Szczesna-Cordary D (2020). Insights into myosin regulatory and essential light chains: a focus on their roles in cardiac and skeletal muscle function, development and disease. J Muscle Res Cell Motil.

[53] Lymn RW, Taylor EW (1970). Transient state phosphate production in the hydrolysis of nucleoside triphosphates by myosin. Biochemistry.

[54] Lymn RW, Taylor EW (1971). Mechanism of adenosine triphosphate hydrolysis by actomyosin. Biochemistry.

[55] Heissler SM, Manstein DJ (2011). Comparative kinetic and functional characterization of the motor domains of human nonmuscle myosin-2 C isoforms. J Biol Chem.

[56] Wang F, Kovacs M, Hu A, Limouze J, Harvey EV, Sellers JR (2003). Kinetic mechanism of non-muscle myosin IIB: functional adaptations for tension generation and maintenance. J Biol Chem.

[57] Kovacs M, Wang F, Hu A, Zhang Y, Sellers JR (2003). Functional divergence of human cytoplasmic myosin II: kinetic characterization of the non-muscle IIA isoform. J Biol Chem.

[58] Fischer S, Windshugel B, Horak D, Holmes KC, Smith JC (2005). Structural mechanism of the recovery stroke in the myosin molecular motor. Proc Natl Acad Sci U S A.

[59] Nagy A, Takagi Y, Billington N, Sun SA, Hong DK, Homsher E, Wang A, Sellers JR (2013). Kinetic characterization of nonmuscle myosin IIb at the single molecule level. J Biol Chem.

[60] Robert-Paganin J, Pylypenko O, Kikuti C, Sweeney HL, Houdusse A (2020). Force generation by Myosin Motors: a structural perspective. Chem Rev.

[61] von der Ecken J, Heissler SM, Pathan-Chhatbar S, Manstein DJ, Raunser S (2016). Cryo-EM structure of a human cytoplasmic actomyosin complex at near-atomic resolution. Nature.

[62] Rosenfeld SS, Xing J, Chen LQ, Sweeney HL (2003). Myosin IIb is unconventionally conventional. J Biol Chem.

[63] Heissler SM, Sellers JR (2016). Kinetic adaptations of myosins for their Diverse Cellular functions. Traffic.

[64] Bloemink MJ, Geeves MA (2011). Shaking the myosin family tree: biochemical kinetics defines four types of myosin motor. Semin Cell Dev Biol.

[65] Melli L, Billington N, Sun SA, Bird JE, Nagy A, Friedman TB, Takagi Y, Sellers JR (2018) Bipolar filaments of human nonmuscle myosin 2-A and 2-B have distinct motile and mechanical properties. Elife 7. 10.7554/eLife.32871 10.7554/eLife.32871 PMC5829915 29419377

[66] Norstrom MF, Smithback PA, Rock RS (2010). Unconventional processive mechanics of non-muscle myosin IIB. J Biol Chem.

[67] Marston SB, Taylor EW (1980). Comparison of the myosin and actomyosin ATPase mechanisms of the four types of vertebrate muscles. J Mol Biol.

[68] Nyitrai M, Geeves MA (2004). Adenosine diphosphate and strain sensitivity in myosin motors. Philos Trans R Soc Lond B Biol Sci.

[69] Heissler SM, Liu X, Korn ED, Sellers JR (2013). Kinetic characterization of the ATPase and actin-activated ATPase activities of Acanthamoeba castellanii myosin-2. J Biol Chem.

[70] Vitriol EA, Quintanilla MA, Tidei JJ, Troughton LD, Cody A, Cisterna BA, Jane ML, Oakes PW, Beach JR (2023). Nonmuscle myosin 2 filaments are processive in cells. Biophys J.

[71] Straight AF, Cheung A, Limouze J, Chen I, Westwood NJ, Sellers JR, Mitchison TJ (2003). Dissecting temporal and spatial control of cytokinesis with a myosin II inhibitor. Science.

[72] Kepiro M, Varkuti BH, Vegner L, Voros G, Hegyi G, Varga M, Malnasi-Csizmadia A (2014). Para-nitroblebbistatin, the non-cytotoxic and photostable myosin II inhibitor. Angew Chem Int Ed Engl.

[73] Ramamurthy B, Yengo CM, Straight AF, Mitchison TJ, Sweeney HL (2004). Kinetic mechanism of blebbistatin inhibition of nonmuscle myosin IIb. Biochemistry.

[74] Limouze J, Straight AF, Mitchison T, Sellers JR (2004). Specificity of blebbistatin, an inhibitor of myosin II. J Muscle Res Cell Motil.

[75] Kovacs M, Toth J, Hetenyi C, Malnasi-Csizmadia A, Sellers JR (2004). Mechanism of blebbistatin inhibition of myosin II. J Biol Chem.

[76] Nishimura Y, Shi S, Zhang F, Liu R, Takagi Y, Bershadsky AD, Viasnoff V, Sellers JR (2021) The formin inhibitor SMIFH2 inhibits members of the myosin superfamily. J Cell Sci 134. 10.1242/jcs.253708 10.1242/jcs.253708 PMC8121067 33589498

[77] Chinthalapudi K, Heissler SM, Preller M, Sellers JR, Manstein DJ (2017) Mechanistic insights into the active site and allosteric communication pathways in human nonmuscle myosin-2C, *Elife* 6. 10.7554/eLife.32742 10.7554/eLife.32742 PMC5749951 29256864

[78] Munnich S, Pathan-Chhatbar S, Manstein DJ (2014). Crystal structure of the rigor-like human non-muscle myosin-2 motor domain. FEBS Lett.

[79] Geeves MA, Holmes KC (1999). Structural mechanism of muscle contraction. Annu Rev Biochem.

[80] Kull FJ, Endow SA (2013). Force generation by kinesin and myosin cytoskeletal motor proteins. J Cell Sci.

[81] Preller M, Manstein DJ (2013). Myosin structure, allostery, and mechano-chemistry. Structure.

[82] Furch M, Fujita-Becker S, Geeves MA, Holmes KC, Manstein DJ (1999). Role of the salt-bridge between switch-1 and switch-2 of Dictyostelium myosin. J Mol Biol.

[83] Reubold TF, Eschenburg S, Becker A, Kull FJ, Manstein DJ (2003). A structural model for actin-induced nucleotide release in myosin. Nat Struct Biol.

[84] Holmes KC, Geeves MA (2000). The structural basis of muscle contraction. Philos Trans R Soc Lond B Biol Sci.

[85] Varkuti BH, Yang Z, Kintses B, Erdelyi P, Bardos-Nagy I, Kovacs AL, Hari P, Kellermayer M, Vellai T, Malnasi-Csizmadia A (2012). A novel actin binding site of myosin required for effective muscle contraction. Nat Struct Mol Biol.

[86] Li Y, Lalwani AK, Mhatre AN (2008). Alternative splice variants of MYH9. DNA Cell Biol.

[87] Takahashi M, Kawamoto S, Adelstein RS (1992). Evidence for inserted sequences in the head region of nonmuscle myosin specific to the nervous system. Cloning of the cDNA encoding the myosin heavy chain-B isoform of vertebrate nonmuscle myosin. J Biol Chem.

[88] Kelley CA, Takahashi M, Yu JH, Adelstein RS (1993). An insert of seven amino acids confers functional differences between smooth muscle myosins from the intestines and vasculature. J Biol Chem.

[89] Jana SS, Kim KY, Mao J, Kawamoto S, Sellers JR, Adelstein RS (2009). An alternatively spliced isoform of non-muscle myosin II-C is not regulated by myosin light chain phosphorylation. J Biol Chem.

[90] Kim KY, Kawamoto S, Bao J, Sellers JR, Adelstein RS (2008). The B2 alternatively spliced isoform of nonmuscle myosin II-B lacks actin-activated MgATPase activity and in vitro motility. Biochem Biophys Res Commun.

[91] Ma X, Kawamoto S, Uribe J, Adelstein RS (2006). Function of the neuron-specific alternatively spliced isoforms of nonmuscle myosin II-B during mouse brain development. Mol Biol Cell.

[92] Jana SS, Kawamoto S, Adelstein RS (2006). A specific isoform of nonmuscle myosin II-C is required for cytokinesis in a tumor cell line. J Biol Chem.

[93] Zhang Y, Liu C, Adelstein RS, Ma X (2018). Replacing nonmuscle myosin 2A with myosin 2C1 permits gastrulation but not placenta vascular development in mice. Mol Biol Cell.

[94] Bahler M, Rhoads A (2002). Calmodulin signaling via the IQ motif. FEBS Lett.

[95] Peckham M, Knight PJ (2009). When a predicted coiled coil is really a single alpha-helix, in myosins and other proteins. Soft Matter.

[96] Cohen C, Parry DA (1990). Alpha-helical coiled coils and bundles: how to design an alpha-helical protein. Proteins.

[97] Grewe J, Schwarz US (2020). Mechanosensitive self-assembly of myosin II minifilaments. Phys Rev E.

[98] Ricketson D, Johnston CA, Prehoda KE (2010). Multiple tail domain interactions stabilize nonmuscle myosin II bipolar filaments. Proc Natl Acad Sci U S A.

[99] Korkmaz EN, Taylor KC, Andreas MP, Ajay G, Heinze NT, Cui Q, Rayment I (2016). A composite approach towards a complete model of the myosin rod. Proteins.

[100] Taylor KC, Buvoli M, Korkmaz EN, Buvoli A, Zheng Y, Heinze NT, Cui Q, Leinwand LA, Rayment I (2015). Skip residues modulate the structural properties of the myosin rod and guide thick filament assembly. Proc Natl Acad Sci U S A.

[101] Straussman R, Squire JM, Ben-Ya’acov A, Ravid S (2005). Skip residues and charge interactions in myosin II coiled-coils: implications for molecular packing. J Mol Biol.

[102] Rahmani H, Ma W, Hu Z, Daneshparvar N, Taylor DW, McCammon JA, Irving TC, Edwards RJ, Taylor KA (2021) The myosin II coiled-coil domain atomic structure in its native environment. Proc Natl Acad Sci U S A 118. 10.1073/pnas.2024151118 10.1073/pnas.2024151118 PMC8040620 33782130

[103] Nakasawa T, Takahashi M, Matsuzawa F, Aikawa S, Togashi Y, Saitoh T, Yamagishi A, Yazawa M (2005). Critical regions for assembly of vertebrate nonmuscle myosin II. Biochemistry.

[104] Hodge TP, Cross R, Kendrick-Jones J (1992). Role of the COOH-terminal nonhelical tailpiece in the assembly of a vertebrate nonmuscle myosin rod. J Cell Biol.

[105] Shohet RV, Conti MA, Kawamoto S, Preston YA, Brill DA, Adelstein RS (1989). Cloning of the cDNA encoding the myosin heavy chain of a vertebrate cellular myosin. Proc Natl Acad Sci U S A.

[106] Dulyaninova NG, Bresnick AR (2013). The heavy chain has its day: regulation of myosin-II assembly. Bioarchitecture.

[107] Shutova MS, Svitkina TM (2018). Mammalian nonmuscle myosin II comes in three flavors. Biochem Biophys Res Commun.

[108] Ronen D, Ravid S (2009). Myosin II tailpiece determines its paracrystal structure, filament assembly properties, and cellular localization. J Biol Chem.

[109] Rosenberg MM, Ronen D, Lahav N, Nazirov E, Ravid S, Friedler A (2013). High resolution characterization of myosin IIC protein tailpiece and its effect on filament assembly. J Biol Chem.

[110] Kelley CA, Kawamoto S, Conti MA, Adelstein RS (1991). Phosphorylation of vertebrate smooth muscle and nonmuscle myosin heavy chains in vitro and in intact cells. J Cell Sci Suppl.

[111] Breckenridge MT, Dulyaninova NG, Egelhoff TT (2009). Multiple regulatory steps control mammalian nonmuscle myosin II assembly in live cells. Mol Biol Cell.

[112] Juanes-Garcia A, Chapman JR, Aguilar-Cuenca R, Delgado-Arevalo C, Hodges J, Whitmore LA, Shabanowitz J, Hunt DF, Horwitz AR, Vicente-Manzanares M (2015). A regulatory motif in nonmuscle myosin II-B regulates its role in migratory front-back polarity. J Cell Biol.

[113] Trybus KM, Huiatt TW, Lowey S (1982). A bent monomeric conformation of myosin from smooth muscle. Proc Natl Acad Sci U S A.

[114] Craig R, Smith R, Kendrick-Jones J (1983). Light-chain phosphorylation controls the conformation of vertebrate non-muscle and smooth muscle myosin molecules. Nature.

[115] Alamo L, Wriggers W, Pinto A, Bartoli F, Salazar L, Zhao FQ, Craig R, Padron R (2008). Three-dimensional reconstruction of tarantula myosin filaments suggests how phosphorylation may regulate myosin activity. J Mol Biol.

[116] Liu J, Wendt T, Taylor D, Taylor K (2003). Refined model of the 10S conformation of smooth muscle myosin by cryo-electron microscopy 3D image reconstruction. J Mol Biol.

[117] Billington N, Wang A, Mao J, Adelstein RS, Sellers JR (2013). Characterization of three full-length human nonmuscle myosin II paralogs. J Biol Chem.

[118] Cross RA, Jackson AP, Citi S, Kendrick-Jones J, Bagshaw CR (1988). Active site trapping of nucleotide by smooth and non-muscle myosins. J Mol Biol.

[119] Scholey JM, Taylor KA, Kendrick-Jones J (1980). Regulation of non-muscle myosin assembly by calmodulin-dependent light chain kinase. Nature.

[120] Sellers JR, Pato MD, Adelstein RS (1981). Reversible phosphorylation of smooth muscle myosin, heavy meromyosin, and platelet myosin. J Biol Chem.

[121] Vicente-Manzanares M, Koach MA, Whitmore L, Lamers ML, Horwitz AF (2008). Segregation and activation of myosin IIB creates a rear in migrating cells. J Cell Biol.

[122] Beach JR, Bruun KS, Shao L, Li D, Swider Z, Remmert K, Zhang Y, Conti MA, Adelstein RS, Rusan NM, Betzig E, Hammer JA (2017). Actin dynamics and competition for myosin monomer govern the sequential amplification of myosin filaments. Nat Cell Biol.

[123] Heissler SM, Arora AS, Billington N, Sellers JR, Chinthalapudi K (2021). Cryo-EM structure of the autoinhibited state of myosin-2. Sci Adv.

[124] Yang S, Tiwari P, Lee KH, Sato O, Ikebe M, Padron R, Craig R (2020). Cryo-EM structure of the inhibited (10S) form of myosin II. Nature.

[125] Scarff CA, Carrington G, Casas-Mao D, Chalovich JM, Knight PJ, Ranson NA, Peckham M (2020). Structure of the shutdown state of myosin-2. Nature.

[126] Kaufmann TL, Schwarz US (2020). Electrostatic and bending energies predict staggering and splaying in nonmuscle myosin II minifilaments. PLoS Comput Biol.

[127] Liu X, Billington N, Shu S, Yu SH, Piszczek G, Sellers JR, Korn ED (2017). Effect of ATP and regulatory light-chain phosphorylation on the polymerization of mammalian nonmuscle myosin II. Proc Natl Acad Sci U S A.

[128] Verkhovsky AB, Borisy GG (1993). Non-sarcomeric mode of myosin II organization in the fibroblast lamellum. J Cell Biol.

[129] Niederman R, Pollard TD (1975). Human platelet myosin. II. In vitro assembly and structure of myosin filaments. J Cell Biol.

[130] Adelstein RS, Pollard TD, Kuehl WM (1971). Isolation and characterization of myosin and two myosin fragments from human blood platelets. Proc Natl Acad Sci U S A.

[131] Koenderink GH, Paluch EK (2018). Architecture shapes contractility in actomyosin networks. Curr Opin Cell Biol.

[132] Shutova MS, Asokan SB, Talwar S, Assoian RK, Bear JE, Svitkina TM (2017). Self-sorting of nonmuscle myosins IIA and IIB polarizes the cytoskeleton and modulates cell motility. J Cell Biol.

[133] Taneja N, Bersi MR, Baillargeon SM, Fenix AM, Cooper JA, Ohi R, Gama V, Merryman WD, Burnette DT (2020). Precise tuning of cortical contractility regulates cell shape during Cytokinesis. Cell Rep.

[134] Weissenbruch K, Fladung M, Grewe J, Baulesch L, Schwarz US, Bastmeyer M (2022). Nonmuscle myosin IIA dynamically guides regulatory light chain phosphorylation and assembly of nonmuscle myosin IIB. Eur J Cell Biol.

[135] Choi CK, Vicente-Manzanares M, Zareno J, Whitmore LA, Mogilner A, Horwitz AR (2008). Actin and alpha-actinin orchestrate the assembly and maturation of nascent adhesions in a myosin II motor-independent manner. Nat Cell Biol.

[136] Vicente-Manzanares M, Zareno J, Whitmore L, Choi CK, Horwitz AF (2007). Regulation of protrusion, adhesion dynamics, and polarity by myosins IIA and IIB in migrating cells. J Cell Biol.

[137] Lombardo AT, Mitchell CAR, Zaman R, McDermitt DJ, Bretscher A (2024) ARHGAP18-ezrin functions as an autoregulatory module for RhoA in the assembly of distinct actin-based structures. Elife 13. 10.7554/eLife.83526 10.7554/eLife.83526 PMC10830128 38193818

[138] Zaman R, Lombardo A, Sauvanet C, Viswanatha R, Awad V, Bonomo LE, McDermitt D, Bretscher A (2021) Effector-mediated ERM activation locally inhibits RhoA activity to shape the apical cell domain. J Cell Biol 220. 10.1083/jcb.202007146 10.1083/jcb.202007146 PMC8185690 33836044

[139] Chinowsky CR, Pinette JA, Meenderink LM, Lau KS, Tyska MJ (2020). Nonmuscle myosin-2 contractility-dependent actin turnover limits the length of epithelial microvilli. Mol Biol Cell.

[140] Fenix AM, Taneja N, Buttler CA, Lewis J, Van Engelenburg SB, Ohi R, Burnette DT (2016). Expansion and concatenation of non-muscle myosin IIA filaments drive cellular contractile system formation during interphase and mitosis. Mol Biol Cell.

[141] Svitkina TM, Verkhovsky AB, McQuade KM, Borisy GG (1997). Analysis of the actin-myosin II system in fish epidermal keratocytes: mechanism of cell body translocation. J Cell Biol.

[142] Ebrahim S, Fujita T, Millis BA, Kozin E, Ma X, Kawamoto S, Baird MA, Davidson M, Yonemura S, Hisa Y, Conti MA, Adelstein RS, Sakaguchi H, Kachar B (2013). NMII forms a contractile transcellular sarcomeric network to regulate apical cell junctions and tissue geometry. Curr Biol.

[143] Dasbiswas K, Hu S, Bershadsky AD, Safran SA (2019). Registry kinetics of myosin motor stacks driven by Mechanical Force-Induced actin turnover. Biophys J.

[144] Hu S, Grobe H, Guo Z, Wang YH, Doss BL, Pan M, Ladoux B, Bershadsky AD, Zaidel-Bar R (2019). Reciprocal regulation of actomyosin organization and contractility in nonmuscle cells by tropomyosins and alpha-actinins. Mol Biol Cell.

[145] Yu-Kemp HC, Szymanski RA, Cortes DB, Gadda NC, Lillich ML, Maddox AS, Peifer M (2022) Micron-scale supramolecular myosin arrays help mediate cytoskeletal assembly at mature adherens junctions. J Cell Biol 221. 10.1083/jcb.202103074 10.1083/jcb.202103074 PMC8614156 34812842

[146] Pasapera AM, Heissler SM, Eto M, Nishimura Y, Fischer RS, Thiam HR, Waterman CM (2022). MARK2 regulates directed cell migration through modulation of myosin II contractility and focal adhesion organization. Curr Biol.

[147] Aguilar-Cuenca R, Llorente-Gonzalez C, Chapman JR, Talayero VC, Garrido-Casado M, Delgado-Arevalo C, Millan-Salanova M, Shabanowitz J, Hunt DF, Sellers JR, Heissler SM, Vicente-Manzanares M (2020). Tyrosine Phosphorylation of the Myosin Regulatory Light Chain Controls Non-muscle Myosin II Assembly and function in migrating cells. Curr Biol.

[148] Ikebe M, Hartshorne DJ, Elzinga M (1986). Identification, phosphorylation, and dephosphorylation of a second site for myosin light chain kinase on the 20,000-dalton light chain of smooth muscle myosin. J Biol Chem.

[149] Umemoto S, Bengur AR, Sellers JR (1989). Effect of multiple phosphorylations of smooth muscle and cytoplasmic myosins on movement in an in vitro motility assay. J Biol Chem.

[150] Vicente-Manzanares M, Horwitz AR (2010). Myosin light chain mono- and di-phosphorylation differentially regulate adhesion and polarity in migrating cells. Biochem Biophys Res Commun.

[151] Taneja N, Baillargeon SM, Burnette DT (2021). Myosin light chain kinase-driven myosin II turnover regulates actin cortex contractility during mitosis. Mol Biol Cell.

[152] Beach JR, Licate LS, Crish JF, Egelhoff TT (2011). Analysis of the role of Ser1/Ser2/Thr9 phosphorylation on myosin II assembly and function in live cells. BMC Cell Biol.

[153] Nishikawa M, Hidaka H, Adelstein RS (1983). Phosphorylation of smooth muscle heavy meromyosin by calcium-activated, phospholipid-dependent protein kinase. The effect on actin-activated MgATPase activity. J Biol Chem.

[154] Nishikawa M, Sellers JR, Adelstein RS, Hidaka H (1984). Protein kinase C modulates in vitro phosphorylation of the smooth muscle heavy meromyosin by myosin light chain kinase. J Biol Chem.

[155] Feng J, Ito M, Ichikawa K, Isaka N, Nishikawa M, Hartshorne DJ, Nakano T (1999). Inhibitory phosphorylation site for rho-associated kinase on smooth muscle myosin phosphatase. J Biol Chem.

[156] Hartshorne DJ, Ito M, Erdodi F (1998). Myosin light chain phosphatase: subunit composition, interactions and regulation. J Muscle Res Cell Motil.

[157] Kimura K, Ito M, Amano M, Chihara K, Fukata Y, Nakafuku M, Yamamori B, Feng J, Nakano T, Okawa K, Iwamatsu A, Kaibuchi K (1996). Regulation of myosin phosphatase by rho and rho-associated kinase (Rho-kinase). Science.

[158] Eto M, Brautigan DL (2012). Endogenous inhibitor proteins that connect Ser/Thr kinases and phosphatases in cell signaling. IUBMB Life.

[159] Gallis B, Edelman AM, Casnellie JE, Krebs EG (1983). Epidermal growth factor stimulates tyrosine phosphorylation of the myosin regulatory light chain from smooth muscle. J Biol Chem.

[160] Babkoff A, Cohen-Kfir E, Aharon H, Ravid S (2021). Aurora-B phosphorylates the myosin II heavy chain to promote cytokinesis. J Biol Chem.

[161] Ecsedi P, Billington N, Palfy G, Gogl G, Kiss B, Bulyaki E, Bodor A, Sellers JR, Nyitray L (2018). Multiple S100 protein isoforms and C-terminal phosphorylation contribute to the paralog-selective regulation of nonmuscle myosin 2 filaments. J Biol Chem.

[162] Even-Faitelson L, Ravid S (2006). PAK1 and aPKCzeta regulate myosin II-B phosphorylation: a novel signaling pathway regulating filament assembly. Mol Biol Cell.

[163] Murakami N, Chauhan VP, Elzinga M (1998). Two nonmuscle myosin II heavy chain isoforms expressed in rabbit brains: filament forming properties, the effects of phosphorylation by protein kinase C and casein kinase II, and location of the phosphorylation sites. Biochemistry.

[164] Hornbeck PV, Zhang B, Murray B, Kornhauser JM, Latham V, Skrzypek E (2015). PhosphoSitePlus, 2014: mutations, PTMs and recalibrations. Nucleic Acids Res.

[165] Pan BQ, Xie ZH, Hao JJ, Zhang Y, Xu X, Cai Y, Wang MR (2020). PTP1B up-regulates EGFR expression by dephosphorylating MYH9 at Y1408 to promote cell migration and invasion in esophageal squamous cell carcinoma. Biochem Biophys Res Commun.

[166] Liu X, Hong MS, Shu S, Yu S, Korn ED (2013). Regulation of the filament structure and assembly of Acanthamoeba myosin II by phosphorylation of serines in the heavy-chain nonhelical tailpiece. Proc Natl Acad Sci U S A.

[167] Tarrant MK, Cole PA (2009). The chemical biology of protein phosphorylation. Annu Rev Biochem.

[168] Vasquez CG, Heissler SM, Billington N, Sellers JR, Martin AC (2016) Drosophila non-muscle myosin II motor activity determines the rate of tissue folding. Elife 5. 10.7554/eLife.20828 10.7554/eLife.20828 PMC5201417 28035903

[169] Muller M, Diensthuber RP, Chizhov I, Claus P, Heissler SM, Preller M, Taft MH, Manstein DJ (2013). Distinct functional interactions between actin isoforms and nonsarcomeric myosins. PLoS ONE.

[170] Arora AS, Huang HL, Singh R, Narui Y, Suchenko A, Hatano T, Heissler SM, Balasubramanian MK, Chinthalapudi K (2023) Structural insights into actin isoforms. Elife 12. 10.7554/eLife.82015 10.7554/eLife.82015 PMC10072879 36790143

[171] Dong S, Zheng W, Pinkerton N, Hansen J, Tikunova SB, Davis JP, Heissler SM, Kudryashova E, Egelman EH, Kudryashov DS (2022) Photorhabdus luminescens TccC3 toxin targets the Dynamic Population of F-Actin and impairs Cell Cortex Integrity. Int J Mol Sci 23. 10.3390/ijms23137026 10.3390/ijms23137026 PMC9266650 35806028

[172] Barua B, Nagy A, Sellers JR, Hitchcock-DeGregori SE (2014). Regulation of nonmuscle myosin II by tropomyosin. Biochemistry.

[173] Pathan-Chhatbar S, Taft MH, Reindl T, Hundt N, Latham SL, Manstein DJ (2018). Three mammalian tropomyosin isoforms have different regulatory effects on nonmuscle myosin-2B and filamentous beta-actin in vitro. J Biol Chem.

[174] Hundt N, Steffen W, Pathan-Chhatbar S, Taft MH, Manstein DJ (2016). Load-dependent modulation of non-muscle myosin-2A function by tropomyosin 4.2. Sci Rep.

[175] Reindl T, Giese S, Greve JN, Reinke PY, Chizhov I, Latham SL, Mulvihill DP, Taft MH, Manstein DJ (2022). Distinct actin-tropomyosin cofilament populations drive the functional diversification of cytoskeletal myosin motor complexes. iScience.

[176] Hosono Y, Usukura J, Yamaguchi T, Yanagisawa K, Suzuki M, Takahashi T (2012). MYBPH inhibits NM IIA assembly via direct interaction with NMHC IIA and reduces cell motility. Biochem Biophys Res Commun.

[177] Vasioukhin V (2006). Lethal giant puzzle of Lgl. Dev Neurosci.

[178] Li ZH, Spektor A, Varlamova O, Bresnick AR (2003). Mts1 regulates the assembly of nonmuscle myosin-IIA. Biochemistry.

[179] Kiss B, Duelli A, Radnai L, Kekesi KA, Katona G, Nyitray L (2012). Crystal structure of the S100A4-nonmuscle myosin IIA tail fragment complex reveals an asymmetric target binding mechanism. Proc Natl Acad Sci U S A.

[180] Elliott PR, Irvine AF, Jung HS, Tozawa K, Pastok MW, Picone R, Badyal SK, Basran J, Rudland PS, Barraclough R, Lian LY, Bagshaw CR, Kriajevska M, Barsukov IL (2012). Asymmetric mode of ca(2)(+)-S100A4 interaction with nonmuscle myosin IIA generates nanomolar affinity required for filament remodeling. Structure.

[181] Chen PW, Jian X, Heissler SM, Le K, Luo R, Jenkins LM, Nagy A, Moss J, Sellers JR, Randazzo PA (2016). The Arf GTPase-activating protein, ASAP1, binds nonmuscle myosin 2A to Control Remodeling of the Actomyosin Network. J Biol Chem.

[182] Cordenonsi M, D’Atri F, Hammar E, Parry DA, Kendrick-Jones J, Shore D, Citi S (1999). Cingulin contains globular and coiled-coil domains and interacts with ZO-1, ZO-2, ZO-3, and myosin. J Cell Biol.

[183] Rouaud F, Huang W, Flinois A, Jain K, Vasileva E, Di Mattia T, Mauperin M, Parry DAD, Dugina V, Chaponnier C, Mean I, Montessuit S, Mutero-Maeda A, Yan J, Citi S (2023) Cingulin and paracingulin tether myosins-2 to junctions to mechanoregulate the plasma membrane. J Cell Biol 222. 10.1083/jcb.202208065 10.1083/jcb.202208065 PMC10202830 37204781

[184] Harris AR, Jreij P, Fletcher DA (2018). Mechanotransduction by the actin cytoskeleton: converting mechanical stimuli into biochemical signals. Annu Rev Biophys.

[185] Surcel A, Robinson DN (2019). Meddling with myosin’s mechanobiology in cancer. Proc Natl Acad Sci U S A.

[186] Pasapera AM, Plotnikov SV, Fischer RS, Case LB, Egelhoff TT, Waterman CM (2015). Rac1-dependent phosphorylation and focal adhesion recruitment of myosin IIA regulates migration and mechanosensing. Curr Biol.

[187] Vicente-Manzanares M, Newell-Litwa K, Bachir AI, Whitmore LA, Horwitz AR (2011). Myosin IIA/IIB restrict adhesive and protrusive signaling to generate front-back polarity in migrating cells. J Cell Biol.

[188] Greenberg MJ, Arpag G, Tuzel E, Ostap EM (2016). A perspective on the role of Myosins as Mechanosensors. Biophys J.

[189] Kovacs M, Thirumurugan K, Knight PJ, Sellers JR (2007). Load-dependent mechanism of nonmuscle myosin 2. Proc Natl Acad Sci U S A.

[190] Schiffhauer ES, Luo T, Mohan K, Srivastava V, Qian X, Griffis ER, Iglesias PA, Robinson DN (2016). Mechanoaccumulative elements of the mammalian actin Cytoskeleton. Curr Biol.

[191] Surcel A, Schiffhauer ES, Thomas DG, Zhu Q, DiNapoli KT, Herbig M, Otto O, West-Foyle H, Jacobi A, Krater M, Plak K, Guck J, Jaffee EM, Iglesias PA, Anders RA, Robinson DN (2019). Targeting Mechanoresponsive proteins in Pancreatic Cancer: 4-Hydroxyacetophenone blocks Dissemination and Invasion by activating MYH14. Cancer Res.

[192] Ma X, Kovacs M, Conti MA, Wang A, Zhang Y, Sellers JR, Adelstein RS (2012). Nonmuscle myosin II exerts tension but does not translocate actin in vertebrate cytokinesis. Proc Natl Acad Sci U S A.

[193] Walklate J, Ujfalusi Z, Geeves MA (2016). Myosin isoforms and the mechanochemical cross-bridge cycle. J Exp Biol.

[194] Luo T, Mohan K, Iglesias PA, Robinson DN (2013). Molecular mechanisms of cellular mechanosensing. Nat Mater.

[195] Morano I, Chai GX, Baltas LG, Lamounier-Zepter V, Lutsch G, Kott M, Haase H, Bader M (2000). Smooth-muscle contraction without smooth-muscle myosin. Nat Cell Biol.

[196] Jansen KA, Atherton P, Ballestrem C (2017). Mechanotransduction at the cell-matrix interface. Semin Cell Dev Biol.

[197] Ashkenazy H, Abadi S, Martz E, Chay O, Mayrose I, Pupko T, Ben-Tal N (2016). ConSurf 2016: an improved methodology to estimate and visualize evolutionary conservation in macromolecules. Nucleic Acids Res.

